# Corynebacteria as a cause of pulmonary infection: a case series and literature review

**DOI:** 10.1186/s41479-018-0054-5

**Published:** 2018-10-05

**Authors:** Katharine Yang, Robert L. Kruse, Weijie V. Lin, Daniel M. Musher

**Affiliations:** 1 Baylor College of Medicine, Houston, TX 77030 USA; 2 Infectious Disease Section, Michael E. DeBakey Veterans Affairs Medical Center, 2002 Holcombe Boulevard, Houston, TX 77030 USA

**Keywords:** Pneumonia, Corynebacteria, Diphtheroids, Normal respiratory flora

## Abstract

**Background:**

In most cases of community-acquired pneumonia (CAP), an etiologic agent is not determined; the most common report from the microbiological evaluation of sputum cites “normal respiratory flora.” Non-diphtheria *Corynebacterium* spp.*,* a component of this flora, is commonly viewed as a contaminant, but it may be the cause of pneumonia and the frequency with which it causes CAP may be underestimated.

**Case presentations:**

This report present 3 cases of CAP in which *Corynebacterium* spp. was clearly the predominant isolate; identification was confirmed by matrix-assisted laser desorption ionization time of flight (MALDI-TOF) mass spectrometry. Two cases were caused by *C. propinquum* and one by *C. striatum.* Two patients had a tracheostomy and one was on hemodialysis. Patients who received an appropriate antibiotic responded well.

**Conclusion:**

When identified as the predominant isolate in sputum from a patient with CAP, *Corynebacterium* spp. should be considered as a potential cause of the infection. In cases with patients who have compromised airway clearance or who are immunocompromised, microaspiration may be responsible. While some *Corynebacterium* spp. are suspectible to antibiotics usually prescribed for CAP, others are susceptible only to vancomycin or aminoglycosides. Vancomycin is thus the appropriate empiric antibiotic, pending speciation and susceptibility test results. The number of reported cases with result of antibiotic susceptibility testing, however, remains limited, and further investigation is needed. Non-diphtheria *Corynebacterium* spp. represent a noteworthy clinical cause of pneumonia. Identification by Gram stain and as a predominant organism on culture demands careful consideration for management.

## Background

Pneumonia is an important cause of medical morbidity and mortality worldwide; pneumonia and influenza are listed as the 8th leading cause of death in the United States, with 57,062 deaths in 2015 [1]. Well-recognized and common causes of community-acquired pneumonia (CAP) in adults include bacteria such as *Streptococcus pneumoniae, Haemophilus influenzae*, and *Staphylococcus aureus*, and viruses such as influenza virus, respiratory syncytial virus, human metapneumovirus, and parainfluenza virus. Intensive recent investigations have failed to identify a causative organism in 50–62% of patients hospitalized for CAP [2, 3].

The most common laboratory report for sputum samples submitted for microbiological study is “mixed respiratory flora.” Pneumonia caused by *S. pneumoniae* is thought to result from unrecognized microaspiration of pneumococci that have colonized the nasopharynx [4]. The authors of the present study have previously hypothesized that unrecognized aspiration of less virulent bacteria that normally inhabit the nasopharynx might also cause pneumonia, especially in persons whose upper airways are bypassed or whose ability to clear aspirated organisms is damaged [5]. The present study examined the possibilities that: (i) careful examination of Gram-stained sputum and culture plates may reveal a predominant bacterium such as *Corynebacterium* spp. that are not generally regarded as a pulmonary pathogen; (ii) quantitative cultures may document a high concentration of these bacteria in sputum; and (iii) these organisms are in fact the cause of some cases of pneumonia.

*Corynebacterium* spp. Gram-positive bacilli that are often dismissed as contaminants have been reported to cause pneumonia (Table 1). New technologies, such as matrix-assisted laser desorption ionization time of flight (MALDI-TOF) mass spectrometry enable rapid and precise identification of bacterial species [6], and highly sensitive techniques such as polymerase chain reaction (PCR) can help exclude a role for viruses, mycoplasma, and chlamydia. With the advent of these new technologies, there has been greater interest in Corynebacteria*,* and these organisms have been cited as emerging pathogens [7].

**Table 1:** Previously documented cases of *Corynebacterium* pulmonary infection

Organism isolated	No. of cases	Age/Sex	Co-morbid conditions	Diagnosis	Methods of Diagnosis	Mortality N (%)
*C. accolens* [46]	1	72F	DM, myocardial infarction, triple aortic bypass, heart failure	Pneumonia	Bronchoalveolar brush sample. Culture also yielded small numbers of *P. aeruginosa*	1 (100%)
*C. afermentans* [19]	1	27F	HIV	Empyema	Pleural fluid and blood culture positive. Sputum culture reported “normal respiratory flora”	0 (0%)
*C. jeikeium* [10, 22, 23, 47]	5	48F, 28F, 29F, 76M, 77F	2 Acute myelogenous leukemia, 1 Hodgkin’s lymphoma and Acute myelomonocytic leukemia, 1 old pulmonary tuberculosis, and 1 COPD	Pneumonia, (necrosis and hemorrhage in 1 case)	Positive gram stain and culture from 1 post-mortem pneumonia; 4 positive respiratory cultures (including 1 TTA). In 1 case, sputum yielded 10^6^ *C. jeikeium,* > 10^7^ *S. aureus* and 10^3^ *P. aeruginosa* per ml; TTA culture yielded > 10^8^ *C. jeikeium*, 10^4^ *P. aeruginosa*, and 10^3^ *S. aureus*; FNA yielded pure growth of *C. jeikeium*	4 (80%)
*C. macginleyi* [28]	1	73F	Metastatic lung adenocarcinoma, chemotherapy, intubated post-bronchoscopy	Ventilator-associated pneumonia	Sputum culture had 10^5^ CFU/mL *C. macginleyi*	0 (0%)
*C. mucifaciens* [26]	1	51M	DM	Cavitary pneumonia	Not described	0 (0%)
*C. propinquum* [14, 48]	4	72F, 79M, 83M, 7F	2 DM, 1 non-Hodgkin’s MALT lymphoma, 2 Rheumatoid arthritis, 2 COPD, 1 ataxia telangiectasia on inhaled corticosteroids	Pneumonia	3 sputum culture; 1 case with bronchial aspirate and broncho-alveolar lavage yielding only *C. propinquum* and blood culture positive for *S. pneumoniae*	0 (0%)
*C. pseudo-diphtheriticum* [12–14, 33–38, 49–55]	40	91F, 40M, 79F, 70 M, 71M, 68M, 72M, 70F, 74M, 76F, 83M, 75M, 68M, 76M, 65M, 68F, 64M, 67M, 62M, 66M, 50F, 27F, 68F, 29M, 47M, 41M, 24M, 43M, 34M, 33M, 33M, 43M, 34M, 29M, 30M, 80F, 9F, 59M, 46M, 75M	2 CKD, 3 CHF, 10 COPD, 4 DM, 2 asthma, SLE, 1 Adult T-cell lymphotrophic virus Ab+, 1 tracheostomy, 2 lung cancers, 1 epidermoid carcinoma, 2 autoimmune disorder on steroid therapy, 2 intubations, 1 amyotrophic lateral sclerosis, 10 HIV/AIDS, 3 previous tuberculosis, 2 recent *Pneumocystis jiroveci* pneumonia, 1 cystic fibrosis	Pneumonia	15 sputum Gram stains, 2 bronchial secretions, 1 bronchial wash; Cultures also yielded fewer numbers of other bacteria: *S. aureus* (2), *H. influenzae* (1), *S. pneumoniae* (3), *S. maltophila* (1), *P. aeruginosa* (1), *E. cloacae* (1)	4 (10%); 9 status unknown, 27 resolved
*C. pseudotuberculosis* [24, 25]	2	23F, 28M	2 veterinary students (1 known exposure to sick horses)	Pneumonia	TTA or CT guided transthoracic biopsy	0 (0%)
*C. striatum* [14–17, 56–58]	7	68F, 47F, 28M, 27M, 69M, 58M, 69F	1 DM, 2 HIV/AIDS, 1 alcohol use, 1 COPD, 1 heart transplant, 1 RA on prednisone	6 pneumonia, 1 empyema	2 sputum, 2 BAL cultures (4 × 10^3^ CFU/mL and > 10^5^ CFU/mL), 1 bronchoscopy brush sample, 1 empyema, 1 pleural fluid +1 lung tissue culture	1 (14%)
*C. ulcerans* [27, 59]	2	78M, 80F	1 Metastatic squamous cell carcinoma of the lung, 1 HTN	Pneumonia	2 positive sputum cultures, 1 confirmed by BAL	2 (100%)
*C. urealyticum* [60]	1	72M	CHF, COPD	Pneumonia	Transtracheal aspirate	0 (0%)
*C. xerosis* [20]	1	63M	AML	Pneumonia	BAL culture yielded 10^5^ *C. xerosis* /mL	1 (100%)
“Aerobic diphtheroids” [61]	1	68M	Heavy smoking history, dental extractions	Empyema	Pleural fluid culture grew only “diphtheroids”	0 (0%)

*DM* Diabetes Mellitus, *HTN* Hypertension, *HLD* Hyperlipidemia, *HIV* Human Immunodeficiency Virus, *CFU* colony forming units, *COPD* Chronic Obstructive Pulmonary Disease, *RML* Right middle lobe of lung, *TTA* transtracheal aspiration, *FNA* Fine Needle Aspiration, *cx* culture, *MALT* Mucosa-associated lymphoid tissue, *CKD* Chronic kidney disease, *CHF* Congestive heart failure, *SLE* Systemic Lupus Erythematosis, *AIDS* Acquired Immunodeficiency Syndrome, *RA* Rheumatoid arthritis, *BAL* bronchoaveolar lavage

The present study reports on 3 well-documented cases of CAP due to Corynebacteria (2 cases of *C. propinquum*, 1 case of *C. striatum*) that were diagnosed in a 6-month period at a large tertiary care hospital. The study reviews the medical literature on *Corynebacterium* spp. as etiologic agents of pneumonia, with the goal of increasing awareness of the possibility that so-called “normal respiratory flora” may actually be responsible for an as-yet undetermined proportion of cases of CAP.

## Case presentations

### Case 1

**Fig. 1:**
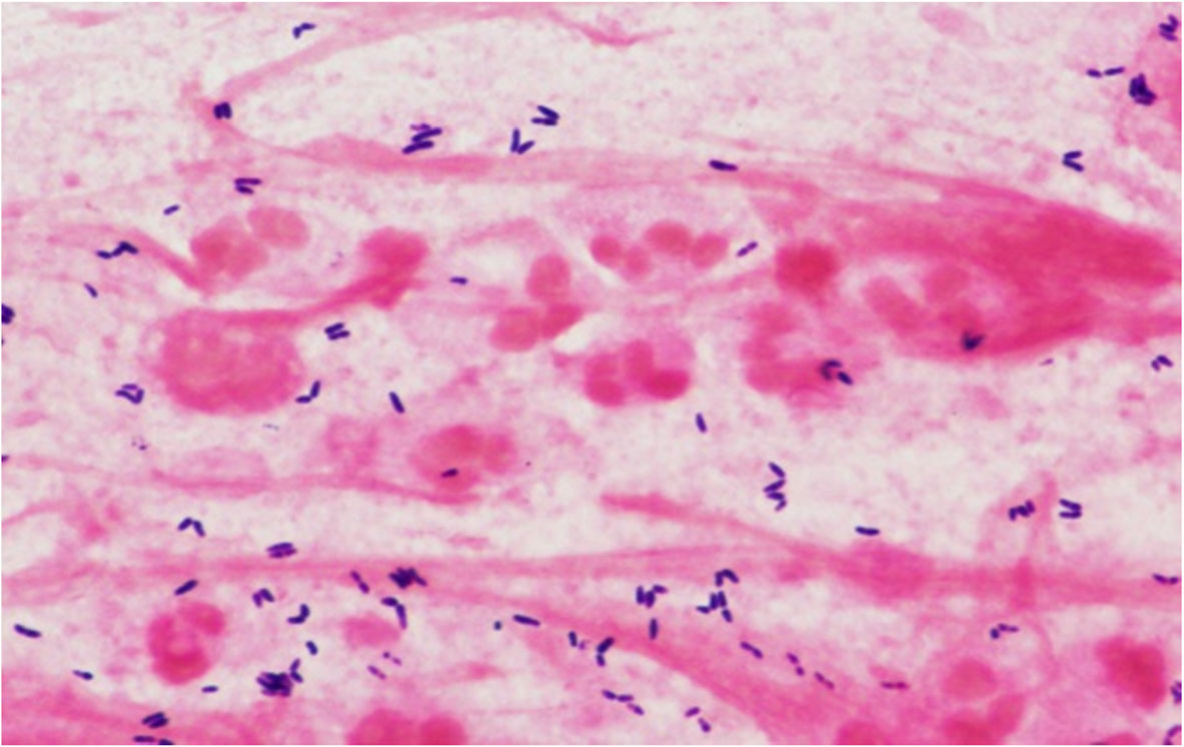
Gram-stain observed in sputum from patient with Corynebacterial pneumonia. A sputum sample collected from the patient in Case 1 depicts Gram-positive bacilli (purple) within and surrounding polymorphonuclear cells (pink). The “club-shaped” appearance pathognomonic for the group “Coryneform” is appreciated

A 75-year-old man on hemodialysis for end-stage renal disease was hospitalized for cough, fever, and altered mental status. His temperature was 103 °F, pulse 68, blood pressure 124/69, respiratory rate 22, and O_2_ saturation 96% on 2 L of O_2_ by nasal cannula. He was lethargic, with bibasilar crackles and a normal cardiac examination. His white blood cell (WBC) count was 20,100 cells/mm^3^ and his plasma procalcitonin was 1.1 ng/mL. Chest X-ray showed bibasilar infiltrates. Microscopic examination of Gram-stained sputum showed profuse polymorphonuclear leukocytes (PMNs) and Gram-positive rods, many of which appeared to be intracellular (Fig. 1). Sputum culture yielded overwhelmingly predominant Corynebacteria*,* with rare *P. aeruginosa*. The Corynebacteria was identified as *C. propinquum* by MALDI-TOF. Blood cultures and viral PCR were negative. The patient was initially treated with vancomycin, cefepime, and metronidazole. Based on the predominance of Corynebacteria with the absence of other bacteria on Gram stain and a negative PCR for respiratory viruses, his pneumonia was attributed to *C. propinquum*, and only vancomycin was continued. He responded and was discharged to complete a 10-day course of linezolid.

### Case 2

A 61-year-old man was found unresponsive post laryngectomy and tracheostomy for laryngeal squamous cell carcinoma. His temperature was 97.8 °F, pulse 99, blood pressure 114/86, and respiratory rate 22. Breath sounds were decreased bilaterally. WBC count was 7200 cells/mm^3^, lactate 1.2 mg/dL. Chest X-ray showed a new left-sided infiltrate. Sputum Gram stain revealed many Gram-positive rods, including those found within PMNs. Sputum yielded many Corynebacteria, identified by MALDI-TOF as *C. propinquum*, and few *S. aureus*. Sputum was liquefied with 2% N-acetyl cysteine and diluted serially; aliquots were cultured on blood agar and the number of colony forming units (CFU) was calculated [8]. The specimen contained 2 × 10^9^ CFU of Corynebacteria and < 10^5^ CFU of *S. aureus* per mL. Blood cultures and viral PCR were negative. The patient was initially given vancomycin, cefepime, and ampicillin; treatment was switched to ampicillin/sulbactam after the *Corynebacterium* was reported susceptible. His mental status rapidly improved, and he was subsequently discharged to complete a 10-day course of amoxicillin/clavulanate.

### Case 3

A 59-year-old male with widely metastatic squamous cell carcinoma of the tongue was admitted for bleeding from his tracheostomy site. His temperature was 99 °F, blood pressure 108/84, pulse 119, respiratory rate 24, and oxygen saturation 82% on room air. He had blood at the tracheostomy site and bibasilar rhonchi. WBC count was 22,000 cells/mm^3^, hemoglobin 11.1 g/dL, and lactate 1.8 mg/dL. Chest X-ray revealed a left upper-lobe infiltrate. Sputum sample showed profuse PMNs and Gram-positive rods. Culture yielded *C. striatum* (confirmed by MALDI-TOF) and few *Escherichia coli*. The patient was placed on comfort care and died 8 days later.

## Discussion

### Epidemiology

Non-diphtheria *Corynebacterium* spp.--commensal flora of the skin, respiratory tract, and mucous membranes [9, 10]-- have been characterized as emerging pathogens [7]. Among reported cases, infections of the respiratory tract are second in frequency only to those of the urinary tract [11]. The literature search revealed 67 reported cases of pulmonary infection due to non-diphtheria *Corynebacterium* spp*.*, summarized in Table 1. Cases include pneumonia (community-acquired, hospital-acquired, or ventilator-associated), necrotizing pneumonia, and empyema, although pneumonia predominated. Implicated *Corynebacterium* spp. include *C. pseudodiphtheriticum* [12, 13], *C. propinquum* [14], *C. striatum* [14–18], *C. afermentans* [19], *C. xerosis* [20], *C. jeikeium* [10, 21–23], *C. pseudotuberculosis* [24, 25], *C. mucifaciens* [26], *C. ulcerans* [27], and *C. macginleyi* [28].

### Microbiology, pathogenesis, and immunity

“Coryneform bacteria” is the umbrella term for the class of bacteria comprised of facultative intracellular, non-spore-forming, catalase-positive, non-acid-fast, non-motile, and irregularly-shaped Gram-positive rods [29]. Based on 16S rRNA sequencing studies, *Corynebacterium* belongs to a subdivision of Gram-positive eubacteria with high guanine-to-cytosine content, which also contains *Arthrobacter, Mycobacterium, Nocardia*, and *Streptomyces* [30]. Coryneform bacteria are pleomorphic throughout their life cycle, and may have thickenings at either end, giving the “coryneform” name, meaning “club-shaped”; they may be clustered, forming shapes that resemble a “V,” “palisades,” or “Chinese letters” [29]. Because of their relation to *C. diphtheriae,* their common name is “diphtheroids.”

Little is known about the virulence factors of the non-diphtheria *Corynebacterium* spp. that may contribute to CAP and other infections [31]. *C. pseudodiphtheriticum,* the species most commonly implicated as a cause of pneumonia, has an ability to invade human epithelial type 2 (HEp-2) cells and survive for 24 h after infection, suggesting a potential mechanism to escape killing by the innate immune system [31]. Invasive strains form biofilms and bind fibrinogen and fibronectin, which may be facilitated by fimbrial subunits [31, 32].

### Predisposing conditions

With the exception of 2 cases of pneumonia due to *C. pseudotuberculosis* in veterinary workers [24, 25], all other reported cases of Corynebacterial pneumonia have occurred in persons whose upper airways are bypassed, whose ability to clear aspirated organisms is damaged, or who have immunocompromising conditions (Table 2). The great majority of patients have had conditions that affect pulmonary clearance, including chronic obstructive pulmonary disease (COPD), cystic fibrosis [33], and previous radiotherapy to the thorax [27]. Some have had procedures that bypass protective mechanisms of the upper airways such as recent endotracheal intubation [28, 34, 35] or laryngectomy [36], as seen in Cases 2 and 3 in the present study. Among patients who are immunocompromised, co-morbidities included HIV infection [17, 37, 38], chemotherapy [10, 28], hematologic malignancies or conditions that compromise PMN function such as diabetes mellitus, alcohol use disorder [16] or end-stage renal disease, as seen in Case 1 in the present study.

**Table 2:** Risk factors in reported cases of Corynebacterial pneumonia

Risk Category	Examples
Decreased clearance / Damaged lung structure	Chronic obstructive pulmonary disease Previous radiation to thorax Cystic fibrosis Heavy smoking history
Bypass of airway protection	Active or recent endotracheal intubation Laryngectomy
Immunodeficiency	Human immunodeficiency virus (HIV) infection Ongoing or recent cancer chemotherapy Conditions that compromise function of PMNs (e.g. poorly controlled diabetes mellitus, end-stage renal disease) Steroid use secondary to autoimmune disease Immunosuppressive drug use secondary to transplant
Miscellaneous factors	Alcohol use disorder
Environmental	Exposure to sick animals (*C. pseudotuberculosis* only*)*

### Clinical description

The presentation of pneumonia due to Corynebacteria does not differ from that due to bacterial pneumonia of any other cause: cough, fever, and altered mental status are usually present, as observed in the present study’s 3 cases. Cases of community-acquired or ventilator-associated pneumonia, cavitary pneumonia, empyema, and bacteremia have been described (Table 1). Only 5 (7.5%) of the 67 reported patients with Corynebacterial pneumonia have been bacteremic with Corynebacteria. This suggests that blood cultures may be of minimal utility for confirming any sputum findings. Pneumonia caused by the quintessential pulmonary pathogen, *S. pneumoniae,* is bacteremic in no more than approximately 25% of cases [39], and the rate of bacteremia is far lower in pneumonia due to less pathogenic organisms, such as nontypable *H. influenzae* [40] or *Moraxella catarrhalis* [41].

### Laboratory diagnosis

Given the limited utility of blood cultures in diagnosing Corynebacterial pneumonia, diagnosis requires a multifaceted approach. In most previous case reports, diagnosis has been based on microscopic examination of a Gram-stained sputum and careful examination of culture plates [42]. In the 3 cases discussed in this report, the sputum Gram stain showed profuse numbers of Corynebacteria*.* In 12 of the previously reported cases, quantitative studies were carried out on sputum, bronchoalveolar lavage samples, or protected bronchial brush samples (Table 1) with few or no colonies of other potentially pathogenic bacteria, as in Case 2 in which quantitative sputum culture revealed 1.2 × 10^9^ CFU/mL. Pneumonia due to Corynebacteria may occur far more commonly than has been reported, because the finding of these organisms is usually dismissed as “normal mouth flora” or “normal respiratory flora.”

MALDI-TOF is useful in providing precise speciation, as was employed in the 3 cases discussed in this report. An analysis of 83 Corynebacterial samples compared MALDI-TOF with API Coryne V2.0 (bioMérieux, Marcy l’Etoile, France), which is the method that has been most widely used to speciate Corynebacteria in the past few decades. Identification matched in 73 of 83 (88%) samples [43]. 16S rRNA sequencing was used to resolve the discrepancy in the remaining 10 samples. All 10 matched with MALDI-TOF’s identification, which demonstrates the superiority of this methodology [43, 44].

### Management

*Corynebacterium* spp. that have been identified as causes of pneumonia or empyema are delineated in Table 1. *C. pseudodiptheriticum,* the cause of about two-thirds of reported Corynebacterial pneumonia cases, is susceptible to penicillin, cephalosporins, and vancomycin, but resistant to macrolides, clindamycin, trimethoprim/sulfamethoxazole, quinolones, and/or rifampin (Table 3). Some species, including *C. jeikeium*, *C. macginleyi*, and *C. xerosis*, are resistant to all common antibiotics except glycopeptides such as vancomycin (Table 3). Intermediately resistant organisms such as *C. propinquum* and *C. striatum,* implicated in the present study’s 3 cases, are also consistently susceptible to vancomycin and ampicillin, but variably susceptible to penicillin and cephalosporins, and commonly resistant to macrolides, clindamycin, trimethoprim-sulfamethoxazole, fluoroquinolines, and rifampin. The other reported *Corynebacterium* spp.—*C. accolens, C. afermentans, C. mucifaciens, C. pseudotuberculosis, C. urealyticum, and C. ulcerans—*had only 1–2 case reports each, and/or did not include comprehensive susceptibility reports. These cases are summarized in Table 3. Although only one pneumonia case report [17] included susceptibility to linezolid or tigecycline, a prospective study analyzing infections due to Corynebacteria from a variety of sites revealed that all isolates—the majority being *C. amycolatum, C. accolens, C. minutissimum,* and *C. glucuronolyticum* (strains that are not regularly implicated in pneumonia)—were susceptible to linezolid and tigecycline [45].

**Table 3:** Antibiotic susceptibilities of *Corynebacterium* spp. identified as causes of pulmonary infection

	**Antibiotic**													
**Organism**	**PCN**	**AMX**	**AMC**	**AMP**	**CEF**	**CFZ**	**CXM**	**CRO**	**CTX**	**SXT**	**ERY**	**AZM**	**CLI**	**CIP**
*C. accolens* (*n* = 1)	100		100						100	0	0		0	
*C. afermentans* (*n* = 1)										100				
*C. jeikeium* (*n* = 5)	0			0		0				0	0		0	
*C. macginleyi* (*n* = 1)	0				0	0	0	0	0					0
*C. mucifaciens* (*n* = 1)	100	100						100						
*C. propinquum* (*n* = 1)	100							100	100				0	
*C. pseudodiphtheriticum* (*n* = 24)	100	100	100	100	100	100	100	100	100	45	5		8	73
*C. pseudotuberculosis* (*n* = 2)	100							100		100	100		100	
*C. striatum* (*n* = 7)	60	100	100	100	67	0	0	0	75	0	50	0	0	0
*C. ulcerans* (*n* = 2)	50			100	100						100	100	50	100
*C. urealyticum* (*n* = 1)	100			100	100									
*C. xerosis* (*n* = 1)	0		0	0	0		0			0	0			0
**Organism**	**LCM**	**RIF**	**GEN**	**TOB**	**VAN**	**LZD**	**IPM**	**AMK**	**DOX**	**TET**	**TEC**	**CHL**	**FOF**	**DAP**
*C. accolens* (*n* = 1)		100	0	0	100			100		0			100	
*C. afermentans* (*n* = 1)					100		100							
*C. jeikeium* (*n* = 5)		100	50		100			100				50		
*C. macginleyi* (*n* = 1)					100									
*C. mucifaciens* (*n* = 1)					100			100						
*C. propinquum* (*n* = 1)					100		100							100
*C. pseudodiphtheriticum* (*n* = 24)	0	75	100	100	100		100	100	100	100	86	27		
*C. pseudotuberculosis* (*n* = 2)		100					100			100				
*C. striatum* (*n* = 7)	100	60	100	100	100	100	80	50		50	100			
*C. ulcerans* (*n* = 2)		100	100		100		100			100		100		
*C. urealyticum* (*n* = 1)										100				
*C. xerosis* (*n* = 1)			0		100			0						

Antibiotic susceptibility for each *Corynebacterium* spp. as reported in all previous cases of Corynebacterial pneumonia (see Table 1 for reference listing) was calculated from number of sensitive isolates as a percentage of the total number of isolates tested with that antibiotic. Blank boxes represent a lack of testing information. The total number of isolates of each species is provided (n). Not all isolates under each organism were tested for the same antibiotics.

*PCN* penicillin, *AMX* amoxicillin, *AMC* amoxicillin-clavulanic acid, *AMP* ampicillin, *CEF* cefalothin, *CFZ* cefazolin, *CXM* cefuroxime, *FOX* cefoxitin, *CRO* ceftriaxone, *CTX* cefotaxime, *OFX* ofloxacin, *SXT* trimethoprim-sulfamethoxazole, *ERY* erythromycin, *AZM* azithromycin, *CLI* clindamycin, *LCM* lincomycin, *RIF* rifampin, *GEN* gentamicin, *TOB* tobramycin, *VAN* vancomycin, *LZD* linezolid, *IPM* Imipenem, *AMK* amikacin, *DOX* doxycycline, *TET* tetracycline, *TEC* teicoplanin, *CHL* chloramphenicol, *FOF* Fosfomycin, *DAP* daptomycin

These results suggest that, when a Gram-stained sputum from a patient with pneumonia reveals Corynebacteria as the predominant isolate, vancomycin should be the initial antibiotic of choice. If MALDI-TOF identifies a species that is likely to be susceptible to other antibiotics, the empiric antibiotic of choice can be revised, or definitive antibiotic treatment can be given based on results of antibiotic susceptibility testing as determined by gradient diffusion strip testing. There is no accepted recommendation for length of treatment for Corynebacterial pneumonia. However, among case reports, treatment time has ranged from 10 days for a case of *C. macginleyi* pneumonia [28], to 8 weeks for a cavitating pneumonia due to *C. jeikeium* [22], to 14 months for pneumonia due to *C. pseudotuberculosis* [25]. It seems reasonable to select a duration of treatment based on the nature of the lung lesion. Infiltrates should respond to the same 7 days of treatment that are used for other bacterial pneumonias, whereas cavitating pneumonia should be treated for longer periods, perhaps until the cavity is closed. In some cases, inadequate responses have been observed in cases of a lung abscess or cavitation, prompting surgical resection [16, 37].

## Conclusions

Although traditionally regarded as part of normal respiratory flora, Corynebacteria has the capacity to cause pneumonia in immunocompromised patients, or in immunocompetent patients who have impaired airway clearance and/or structural damage to the airways. The presentation is similar to that of any other bacterial pneumonia. Such infections may be far more common than has been previously suggested, because the finding of Corynebacteria on Gram stain and culture is likely to lead to a report of “mixed” or “normal respiratory flora.” A correct diagnosis can be made primarily by visualization of large numbers of diphtheroids in microscopic fields that contain > 20 WBC per epithelial cell, especially if the bacteria are located within PMNs, and supported by a culture in which the overwhelmingly predominant organism is Corynebacteria*.* Speciation by MALDI-TOF is increasingly available in clinical laboratories. Initial treatment should be with vancomycin. Speciation may guide antibiotic choice, but definitive therapy should be based on susceptibility testing using gradient diffusion strip testing methodology.

## Abbreviations

CAP: Community-acquired pneumonia
CFU: Colony forming unit
COPD: Chronic obstructive pulmonary disease
HEp-2: Human epithelial type 2
MALDI-TOF: Matrix-assisted laser desorption ionization– time of flight
PMNs: Polymorphonuclear leukocytes
rRNA: Ribosomal RNA
spp.: Species
WBC: White blood cell

## References

[1] National Center for Health Statistics. Health, United States, 2016: With Chartbook on Long-term Trends in Health. Hyattsville, MD. 2017. 28910066

[2] Musher DM, Roig IL, Cazares G (2013). Can an etiologic agent be identified in adults who are hospitalized for community-acquired pneumonia: results of a one-year study. J Inf Secur.

[3] Jain S, Self WH, Wunderink RG, Team CES (2015). Community-acquired pneumonia requiring hospitalization. N Engl J Med.

[4] Janoff EN, Musher DM, Bennett JEDR, Blaser MJ (2015). Streptococcus pneumoniae. Mandell, Douglas, and Bennett's principles and practice of infectious diseases.

[5] Musher DM, Abers MS, Bartlett JG (2017). Evolving understanding of the causes of pneumonia in adults, with special attention to the role of pneumococcus. Clin Infect Dis.

[6] Sloan A, Wang G, Cheng K (2017). Traditional approaches versus mass spectrometry in bacterial identification and typing. Clin Chim Acta.

[7] Harrington AT, Clarridge Iii JE, Mahlen SD, Sussman M, Liu D, Poxton I, Schwartzman J (2015). Chapter 91 - Corynebacterium spp. as established and emerging respiratory pathogens A2 - Tang, Yi-Wei. Molecular Medical Microbiology.

[8] Thorsteinsson SB, Musher DM, Fagan T (1975). The diagnostic value of sputum culture in acute pneumonia. JAMA.

[9] von Graevenitz A, Punter-Streit V, Riegel P, Funke G (1998). Coryneform bacteria in throat cultures of healthy individuals. J Clin Microbiol.

[10] Waters BL (1989). Pathology of culture-proven JK Corynebacterium pneumonia. An autopsy case report. Am J Clin Pathol.

[11] Camello TC, Souza MC, Martins CA (2009). Corynebacterium pseudodiphtheriticum isolated from relevant clinical sites of infection: a human pathogen overlooked in emerging countries. Lett Appl Microbiol.

[12] Ahmed K, Kawakami K, Watanabe K (1995). Corynebacterium pseudodiphtheriticum: a respiratory tract pathogen. Clin Infect Dis.

[13] Manzella JP, Kellogg JA, Parsey KS (1995). Corynebacterium pseudodiphtheriticum: a respiratory tract pathogen in adults. Clin Infect Dis.

[14] Diez-Aguilar M, Ruiz-Garbajosa P, Fernandez-Olmos A (2013). Non-diphtheriae Corynebacterium species: an emerging respiratory pathogen. Eur J Clin Microbiol Infect Dis.

[15] Creagh R, Saavedra JM, Rodriguez FJ (2000). Pneumonia casued by Corynebacterium striatum in a patient with AIDS. Enferm Infecc Microbiol Clin.

[16] Martinez-Martinez L, Suarez AI, Ortega MC, Rodriguez-Jimenez R (1994). Fatal pulmonary infection caused by Corynebacterium striatum. Clin Infect Dis.

[17] Roig-Rico P, Safont-Gaso P, Marin-Tordera D, Ortiz-De la Tabla V (2011). Corynebacterium striatum pneumonia in an HIV patient. Enferm Infecc Microbiol Clin.

[18] Renom F, Gomila M, Garau M (2014). Respiratory infection by Corynebacterium striatum: epidemiological and clinical determinants. New Microbes New Infect.

[19] Minkin R, Shapiro JM (2004). Corynebacterium afermentans lung abscess and empyema in a patient with human immunodeficiency virus infection. South Med J.

[20] Wallet F, Marquette CH, Courcol RJ (1994). Multiresistant Corynebacterium xerosis as a cause of pneumonia in a patient with acute leukemia. Clin Infect Dis.

[21] Ifantidou AM, Diamantidis MD, Tseliki G (2010). Corynebacterium jeikeium bacteremia in a hemodialyzed patient. Int J Infect Dis.

[22] McNaughton RD, Villanueva RR, Donnelly R (1988). Cavitating pneumonia caused by Corynebacterium group JK. J Clin Microbiol.

[23] Yoshitomi Y, Kohno S, Koga H (1992). Fatal pneumonia caused by Corynebacterium group JK after treatment of Staphylococcus aureus pneumonia. Intern Med.

[24] Keslin MH, McCoy EL, McCusker JJ, Lutch JS (1979). Corynebacterium pseudotuberculosis. A new cause of infectious and eosinophilic pneumonia. Am J Med.

[25] Heggelund L, Gaustad P, Havelsrud OE (2015). Corynebacterium pseudotuberculosis pneumonia in a veterinary student infected during laboratory work. Open Forum Infect Dis.

[26] Djossou F, Bezian MC, Moynet D (2010). Corynebacterium mucifaciens in an immunocompetent patient with cavitary pneumonia. BMC Infect Dis.

[27] Siegel SM, Haile CA (1985). Corynebacterium ulcerans pneumonia. South Med J.

[28] Kebbe J, Mador MJ (2015). Corynebacterium macginleyi: a cause of ventilator associated pneumonia in an immunocompromised patient. Respir Med Case Rep.

[29] Funke G, von Graevenitz A, Clarridge JE, Bernard KA (1997). Clinical microbiology of coryneform bacteria. Clin Microbiol Rev.

[30] Woese CR (1987). Bacterial evolution. Microbiol Rev.

[31] Burkovski A (2015). Corynebacterium pseudodiphtheriticum: putative probiotic, opportunistic infector, emerging pathogen. Virulence.

[32] Souza MC, dos Santos LS, Sousa LP (2015). Biofilm formation and fibrinogen and fibronectin binding activities by Corynebacterium pseudodiphtheriticum invasive strains. Antonie Van Leeuwenhoek.

[33] Bittar F, Cassagne C, Bosdure E (2010). Outbreak of Corynebacterium pseudodiphtheriticum infection in cystic fibrosis patients, France. Emerg Infect Dis.

[34] Carranza Gonzalez R, Tena Gomez D, Prieto Gomez E (2006). Pneumonia by Corynebacterium pseudodiphteriticum: an infection to consider. An Med Interna.

[35] Miller RA, Rompalo A, Coyle MB (1986). Corynebacterium pseudodiphtheriticum pneumonia in an immunologically intact host. Diagn Microbiol Infect Dis.

[36] Chiner E, Arriero JM, Signes-Costa J (1999). Corynebacterium pseudodiphtheriticum pneumonia in an immunocompetent patient. Monaldi Arch Chest Dis.

[37] Gutierrez-Rodero F, Ortiz de la Tabla V, Martinez C (1999). Corynebacterium pseudodiphtheriticum: an easily missed respiratory pathogen in HIV-infected patients. Diagn Microbiol Infect Dis.

[38] Roig P, Lopez MM, Arriero JM (1993). Corynebacterium pseudodiphtheriticum pneumonia in a patient diagnosed with HIV infection. An Med Interna.

[39] Heffron R (1939). Pneumonia with special reference to pneumococcus lobar pneumonia.

[40] Musher DM, Kubitschek KR, Crennan J, Baughn RE (1983). Pneumonia and acute febrile tracheobronchitis due to *Haemophilus influenzae*. Ann Intern Med.

[41] Wallace RJ, Musher DM (1986). In honor of Dr. Sarah Branham, a star is born. The realization of Branhamella catarrhalis as a respiratory pathogen. Chest.

[42] Coyle MB, Lipsky BA (1990). Coryneform bacteria in infectious diseases: clinical and laboratory aspects. Clin Microbiol Rev.

[43] Vila J, Juiz P, Salas C (2012). Identification of clinically relevant Corynebacterium spp., Arcanobacterium haemolyticum, and Rhodococcus equi by matrix-assisted laser desorption ionization-time of flight mass spectrometry. J Clin Microbiol.

[44] Bao R, Gao X, Hu B, Zhou Z (2017). Matrix-assisted laser desorption ionization time-of-flight mass spectrometry: a powerful tool for identification of Corynebacterium species. J Thorac Dis.

[45] Reddy BS, Chaudhury A, Kalawat U (2012). Isolation, speciation, and antibiogram of clinically relevant non-diphtherial Corynebacteria (Diphtheroids). Indian J Med Microbiol.

[46] Losada I, Daza RM, Merino J (1996). Corynebacterium CDC G1: pathogen or colonizer?. Enferm Infecc Microbiol Clin.

[47] Riebel W, Frantz N, Adelstein D, Spagnuolo PJ (1986). Corynebacterium JK: a cause of nosocomial device-related infection. Rev Infect Dis.

[48] Malkocoglu G, Gencer H, Kaya A (2016). Corynebacterium propinquum bronchopneumonia in a child with ataxia telangiectasia. Turk J Pediatr.

[49] Furiasse D, Gasparotto AM, Monterisi A (2016). Pneumonia caused byCorynebacterium pseudodiphtheriticum. Rev Argent Microbiol.

[50] Chudnicka A, Szmygin-Milanowska K, Kieszko R (2003). The role of opportunistic species of Corynebacterium pseudodiphtheriticum in the pathogenesis of CAP (community acquired pneumonia). Ann Univ Mariae Curie Sklodowska Med.

[51] Aspiroz Sancho C, Agustin Berne A, Navarro Pardos C (2002). Pneumonia caused by Corynebacterium pseudodiphteriticum, an entity worth knowing. An Med Interna.

[52] Martaresche C, Fournier PE, Jacomo V (1999). A case of Corynebacterium pseudodiphtheriticum nosocomial pneumonia. Emerg Infect Dis.

[53] Cohen Y, Force G, Gros I (1992). Corynebacterium pseudodiphtheriticum pulmonary infection in AIDS patients. Lancet.

[54] Donaghy M, Cohen J (1983). Pulmonary infection with Corynebacterium hofmannii complicating systemic lupus erythematosus. J Infect Dis.

[55] Nishiyama A, Ishida T, Ito A, Arita M (2013). Bronchopneumonia caused by Corynebacterium pseudodiphtheriticum. Intern Med.

[56] Cowling P, Hall L (1993). Corynebacterium striatum: a clinically significant isolate from sputum in chronic obstructive airways disease. J Inf Secur.

[57] Tarr PE, Stock F, Cooke RH (2003). Multidrug-resistant Corynebacterium striatum pneumonia in a heart transplant recipient. Transpl Infect Dis.

[58] Verma R, Kravitz GR. Corynebacterium striatum empyema and osteomyelitis in a patient with advanced rheumatoid arthritis. BMJ Case Rep. 2016; 10.1136/bcr-2016-214691. 10.1136/bcr-2016-214691 PMC4785426 26944378

[59] Mattos-Guaraldi AL, Sampaio JL, Santos CS (2008). First detection of Corynebacterium ulcerans producing a diphtheria-like toxin in a case of human with pulmonary infection in the Rio de Janeiro metropolitan area, Brazil. Mem Inst Oswaldo Cruz.

[60] Jacobs NF, Perlino CA (1979). “Diphtheroid” pneumonia. South Med J.

[61] Nazemi MM, Musher DM (1973). Empyema due to aerobic diphtheroids following dental extraction. Am Rev Respir Dis.

